# Update on the Coordinated Efforts of Looking After the Health Care Needs of Children and Young People Fleeing the Conflict Zone of Ukraine Presenting to European Emergency Departments—A Joint Statement of the European Society for Emergency Paediatrics and the European Academy of Paediatrics

**DOI:** 10.3389/fped.2022.897803

**Published:** 2022-04-26

**Authors:** Ruud G. Nijman, Silvia Bressan, Julia Brandenberger, Davi Kaur, Kristina Keitel, Ian K. Maconochie, Rianne Oostenbrink, Niccolo Parri, Itai Shavit, Ozlem Teksam, Roberto Velasco, Patrick van de Voorde, Liviana Da Dalt, Ann De Guchtenaere, Adamos A. Hadjipanayis, Robert Ross Russell, Stefano del Torso, Zsolt Bognar, Luigi Titomanlio

**Affiliations:** ^1^Division of Medicine, Department of Paediatric Emergency Medicine, St. Mary's Hospital - Imperial College NHS Healthcare Trust, London, United Kingdom; ^2^Section of Paediatric Infectious Diseases, Department of Infectious Diseases, Faculty of Medicine, Imperial College London, London, United Kingdom; ^3^Centre for Paediatrics and Child Health, Imperial College, London, United Kingdom; ^4^Division of Paediatric Emergency Medicine, Department of Women's and Children's Health, University Hospital of Padova, Padova, Italy; ^5^Edwin S.H. Leong Centre for Healthy Children, University of Toronto, Toronto, ON, Canada; ^6^Division of Pediatric Emergency Medicine, Hospital for Sick Children, University of Toronto, Toronto, ON, Canada; ^7^Division of Paediatric Emergency Medicine, Department of Paediatrics, University Hospital of Bern, Bern, Switzerland; ^8^European Society for Emergency Medicine, Antwerp, Belgium; ^9^Department of Epidemiology and Public Health, Swiss Tropical and Public Health Institute, Basel, Switzerland; ^10^Department of General Paediatrics, Erasmus MC – Sophia, Rotterdam, Netherlands; ^11^Emergency Department & Trauma Center, Ospedale Paediatrico Meyer Firenze, Florence, Italy; ^12^Paediatric Emergency Department, Rambam Health Care Campus, Haifa, Israel; ^13^Division of Paediatric Emergency Medicine, Department of Paediatrics, Hacettepe University School of Medicine, Ankara, Turkey; ^14^Paediatric Emergency Unit, Hospital Universitario Río Hortega, Valladolid, Spain; ^15^Federal Department of Health, Ghent University Hospital, Ghent, Belgium; ^16^Department of Paediatrics, Ghent University, Ghent, Belgium; ^17^Department of Medicine, European University Cyprus, Nicosia, Cyprus; ^18^Cambridge University Hospitals NHS Foundation Trust, Cambridge, United Kingdom; ^19^Primary Care Paediatrician, ChildCare Worldwide OdV, Padova, Italy; ^20^Department of Paediatric Emergency Medicine, Heim Pal National Paediatric Institute, Budapest, Hungary; ^21^Paediatric Emergency Department, Hopital Universitaire Robert-Debre, Paris, France; ^22^Université Paris Cité, INSERM UMR 1141, DHU Protect, Paris, France

**Keywords:** emergency medicine, pediatrics, refugee, infectious diseases, mental health, social medicine, trauma, post-traumatic stress disorder

## Abstract

This joint statement by the European Society for Emergency Paediatrics and European Academy of Paediatrics aims to highlight recommendations for dealing with refugee children and young people fleeing the Ukrainian war when presenting to emergency departments (EDs) across Europe. Children and young people might present, sometimes unaccompanied, with either ongoing complex health needs or illnesses, mental health issues, and injuries related to the war itself and the flight from it. Obstacles to providing urgent and emergency care include lack of clinical guidelines, language barriers, and lack of insight in previous medical history. Children with complex health needs are at high risk for complications and their continued access to specialist healthcare should be prioritized in resettlements programs. Ukraine has one of the lowest vaccination coverages in the Europe, and outbreaks of cholera, measles, diphtheria, poliomyelitis, and COVID-19 should be anticipated. In Ukraine, rates of multidrug resistant tuberculosis are high, making screening for this important. Urgent and emergency care facilities should also prepare for dealing with children with war-related injuries and mental health issues. Ukrainian refugee children and young people should be included in local educational systems and social activities at the earliest opportunity.

## Introduction

The tragic events in Ukraine require an immediate and meaningful response from European societies and non-governmental organizations (NGOs) to safeguard children and guarantee their health care needs are met.

On the one hand this means pathways need to be established to continue essential medical treatment of children with known underlying disease. Examples of this include ongoing treatment for oncological conditions, diabetes mellitus, epilepsy, and cystic fibrosis. Difficulties include differences in treatment regimens and care standards, lack of medical documents, and language barriers. From frontline physicians, this will require a coordinating and facilitating role. On the other, we will also need to anticipate children presenting with illnesses, mental health issues, and injuries related to the war itself and the flight from it. There will be a non-negligible public health risk for spread of communicable diseases, such as COVID-19 and multidrug resistant TB. In time, focus and demands of health care provision will shift as the conflict goes on.

From a European perspective, the war in Ukraine and the stream of refugees resulting from this will be markedly different from other recent global conflicts. Importantly, the proximity of Ukraine on the European continent will lead to an immediate influx of refugees, compared with a delayed influx, often after stays in refugee camps and hazardous journeys, observed with conflicts occurring outside the continent.

It is to be expected that emergency departments (EDs) will function as a first point of access for health systems in many countries, and pediatric emergency clinicians, and other health care professionals looking after acutely unwell children, will need to understand their roles and responsibilities when faced with refugee children and young people from Ukraine. An existing European Academy of Pediatrics (EAP) statement provides extensive and evidence based recommendations for first and follow-up appointments for asymptomatic migrants, including refugee children and young people in Europe (1). In addition, a previous European Society of Emergency Pediatricians (EUSEP) survey explored obstacles and needs for providing emergency care to this vulnerable population (2). This statement builds on this previous work and was written by members of the executive committees and experts of the EUSEP and EAP. The statement highlights recommendations on providing urgent and emergency care to refugee children and young people and inform on ongoing efforts to coordinate and support child health care in response to the Ukrainian war.

## Financial Position

Firstly, guidance should be issued at a national level on the financial renumeration when providing healthcare to this group of patients. National pediatric and emergency medicine societies should advocate for an open, and fee free, access to urgent and emergency care services; individual clinicians can contact their representing organizations and stress the importance of this. The EAP will act as the strategic political voice for families and pediatricians within the European Union to convey the advocacy message coming from the national organizations. As pediatric health care professionals we should be able to provide urgent and emergency care to children without financial reservations or constraints. We feel strongly that this should allow for prescription of first line maintenance drugs of existing medical conditions, such as asthma, epilepsy, and diabetes. Similarly, caregivers might use emergency services if they run out of medical devices, such as feeding tubes, and arrangements should be in place to provide supplies. All this should be offered with careful consideration of privacy and General Data Protection Regulation compliant documentation of person identifiable data.

## Sharing of Resources and Coordinating Complex Health Needs

Working together with NGOs and pediatric societies, we have constructed an open access repository for sharing of resources. This includes a channel on the European Society for Emergency Medicine (EUSEM) Academy platform with educational and teaching videos, as well as guidelines and care toolkits in a variety of languages (Table 1). An example template in English/Ukrainian/Russian for registering details of a refugee child or young person presenting at the ED is available in Appendix 1.

**Table 1:** Selection of resources available.

**Organization**	**Description**	**Website or link**	**Language**
***Resources from Ukraine***			
Ukraine Academy of Paediatric Specialties	Website for parents with information leaflets	https://zrozumilo.org	Cyrillic
Ministry of health Ukraine	Immunization schedule	https://en.moz.gov.ua/vaccinations	English, Cyrillic
***Resources from (inter)national societies***			
European Society for Emergency Medicine	Resources and overview of actions for Ukraine	https://eusem.org/news/eusem-actions-for-ukraine	English
European Academy of Paediatrics	Practical guidelines: Medical care for migrant children in Europe: a practical recommendation for first and follow-up appointments	https://link.springer.com/article/10.1007/s00431-019-03405-9	English
Royal College of Paediatrics and Child Health [UK]	Refugee and unaccompanied asylum-seeking children and young people—guidance for pediatricians	https://www.rcpch.ac.uk/resources/refugee-unaccompanied-asylum-seeking-children-young-people-guidance-paediatricians#age-assessment	English
Deutsche Gesellschaft fur Kinder- und Jugendmedizin [Germany]	Guidance for Infection screening for refugee children and young people	https://dgpi.de/infektiologische-versorgung-von-fluechtlingen-im-kindes-und-jugendalter-in-deutschland/	German
Association Espanola de Pediatria [Spain]	Guidance on immunizations for refugee children and young people	https://vacunasaep.org/documentos/manual/cap-12	Spanish
Nederlandse vereniging voor kindergeneeskunde—expertisegroep Global Child Health [Netherlands]	Guidance on management and infection screening of refugee children and young people	https://www.nvk.nl/over-nvk/gremia/expertisegroepen/expertisegroep?groupid=15302687	Dutch
Haut conseil de la Sante Publique [France]	Guidance on health checks for isolated foreign children	https://www.hcsp.fr/Explore.cgi/avisrapportsdomaine?clefr=753	French
National Center for school crisis and bereavement [US]	Talking to children and teens about the war in Ukraine	https://www.schoolcrisiscenter.org	English
American Academy of Pediatrics—Healthier Together [US]	Talking with kids about war	https://www.healthychildren.org	English
***Resources from non-governmental organisations and charities***			
United Nations High Commissioner for Refugees (UNHCR)	The UN refugee agency—Site for Ukraine situation	https://www.unhcr.org/ua/en	English, Cyrillic
Medicins Sans Frontiers	Medical field guidelines	https://www.msf.org/medical-resources	English
United Nations International Children's Emergency Fund (UNICEF)	Resources for refugee and migrant children in Europe	https://www.unicef.org/eca/emergencies/refugee-and-migrant-children-europe	English
World Health Organisation	Emergency Response Learning & Resource Center—WHO academy	https://express.adobe.com/page/XCHiPbrUNNNFC/	English
World Health Organisation	WHO information on vaccination coverage in Ukraine	https://immunizationdata.who.int/pages/profiles/ukr.html	English
World Health Organisation	Technical guidance on Delivery of Immunization services for refugees and migrants	https://apps.who.int/iris/bitstream/handle/10665/326924/9789289054270-eng.pdf?sequence=1&isAllowed=y	English
WHO—Health Cluster Ukraine	Public health situation analyses (PHSA) for Ukraine	https://www.humanitarianresponse.info/en/operations/ukraine/document/ukraine-public-health-situation-analysis-phsa-ukraine-03032022-eng	English
International committee of the Red Cross	Overview of current ICRC operations in the country	https://www.icrc.org/en/where-we-work/europe-central-asia/ukraine	English
International committee of the Red Cross	Blast trauma care—course manual	https://www.icrc.org/en/publication/4500-blast-trauma-care-course-manual	English
Imperial College London—Centre for blast injury studies	Paediatric Blast Injury Field Manual	https://www.imperial.ac.uk/blast-injury/research/networks/paediatric-blast-injury-field-manual/	English, Other
Doctors of the world	Guidance on creating a refugee friendly and safe medical environment	https://www.doctorsoftheworld.org.uk/what-we-stand-for/supporting-medics/safe-surgeries-initiative/safe-surgeries-toolkit/?nabm=1	English
Freedom from torture	Charity for victims of torture	https://www.freedomfromtorture.org	English
Immunization action coalition [US]	Translations of common childhood infections	https://www.immunize.org/catg.d/p5122.pdf	English
Refugee Council UK	Overview of services available to refugees in UK	https://www.refugeecouncil.org.uk/get-support/services/	English
MedTube	Social eLearning platform for medical professionals	https://medtube.net	English
***Resources from international centres for disease control and prevention***			
Center for Disease Control and Prevention (CDC) [US]	Country specific information on communicable diseases by CDC (US)	https://wwwnc.cdc.gov/travel/destinations/traveler/none/ukraine	English
European Center for Disease Control (ECDC)	Operational public health considerations for the prevention and control of infectious diseases in the context of Russia's aggression toward Ukraine	https://www.ecdc.europa.eu/en/publications-data/operational-public-health-considerations-prevention-and-control-infectious	English
European Center for Disease Control (ECDC)	Infographic: Vaccinations to be offered in the absence of documented evidence of prior vaccination	https://www.ecdc.europa.eu/en/publications-data/infographic-vaccinations-offered-absence-documented-prior-vaccination	English

*Selection of resources available via the EUSEM webpage: https://eusem.org/sections-and-committees/sections/paediatric-section/paediatric-resources-for-ukrainian-support. This list is continuously being updated with new materials*.

EUSEM is also working together with NGOs and national societies in an effort to provide teaching and educational resources to people working in conflict areas in Ukraine and neighboring countries.

Previous survey work found that, traditionally, most refugee children and young people attending EDs had similar medical needs to the local population. Most of these required standard urgent and emergency medicine skills; patients with rare infectious diseases or complex mental health issues represented only a minority of the problems faced (2–5). A recently published review gives a patient-centered summary of key challenges in health care delivery for refugees and migrants, which might aid in preparing local healthcare services (3). Obstacles to providing urgent and emergency care were mostly a lack of clinical guidelines, language barriers, and lack of insight in previous medical history. The latter poses a significant risk for children with complex health needs dependent on continued specialist healthcare and the prescribing of regular medications. Efforts should focus on a coordinated resettlement process for children with acute tertiary healthcare needs, for example by means of expanding the existing United Nations High Commissioner for Refugees (UNHCR) resettlement program. The European Children's Hospitals Organisation (ECHO) and European governments are identifying hospitals across Europe that can provide (inpatient) care to pediatric patients with complex medical conditions. During the relocation process refugee children and young people with complex health needs are at high risk of acute complications of their underlying medical disease, likely to result in presentations to EDs. Inherent issues related to displacement, such as traveling, incomplete family structures, stays in refugee camps or other temporary accommodation, malnourishment, and exposure to communicable diseases, will further complicate the health situation of children with complex health needs.

## Language Barrier and Translation Services

Language barriers and cultural differences have been identified as important barriers to providing urgent and emergency care in EDs (2). This is particularly relevant for consenting and assenting procedures for medical assessments, prescribing of medications or urgent surgical procedures. There will likely be high demand on translation services in the near future, and it might not always be possible to connect to translation services when consulting patients in the ED. Arranging access to certified clinical translators is an important early step for each clinical area providing urgent and emergency care. Information leaflets in Cyrillic are available online (6).

## Immunization Schedule

**Figure 1:**
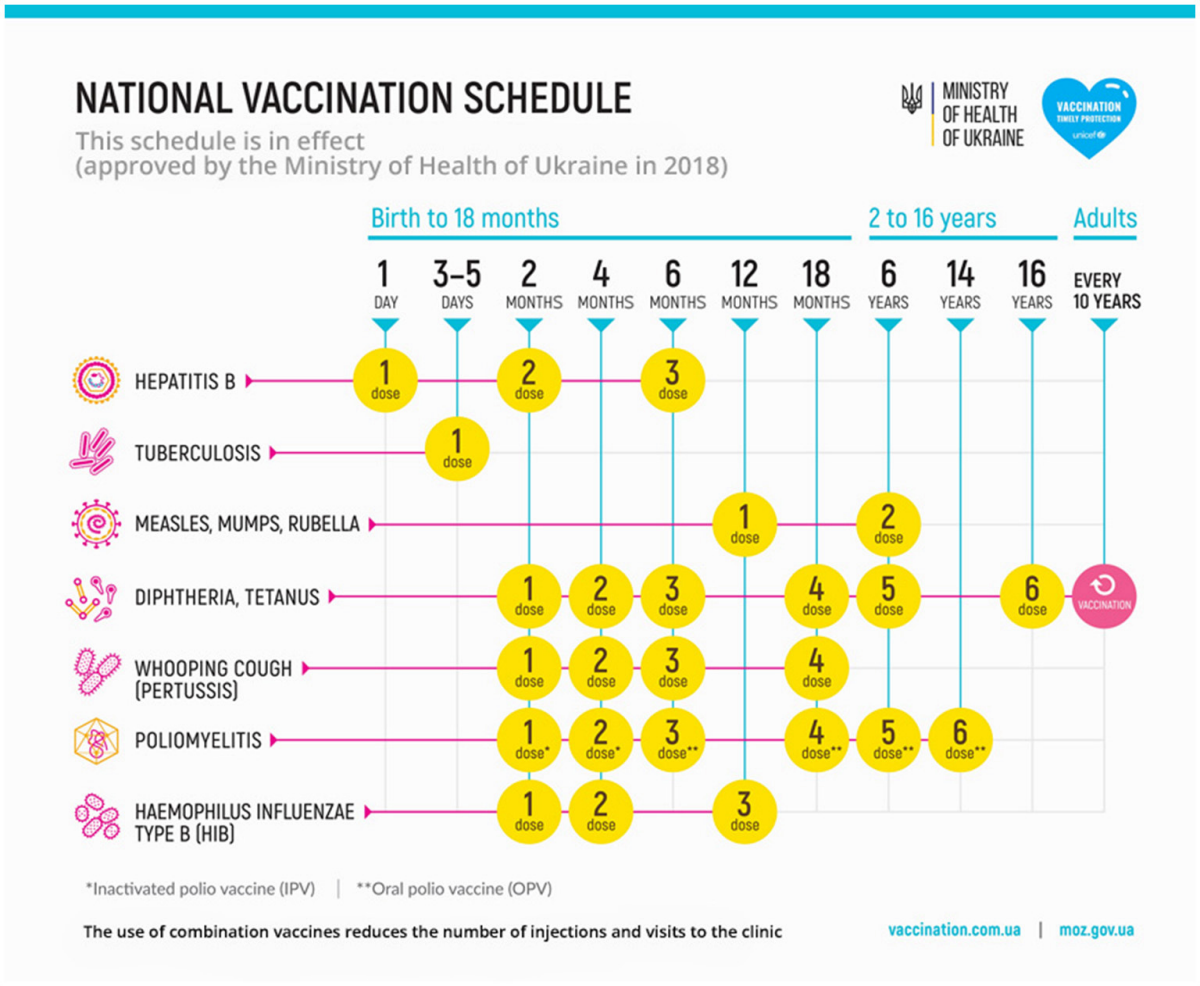
Immunization schedule of Ukraine. source: https://en.moz.gov.ua/vaccinations (accessed 11-03-2022).

The international community should invest in securing that children receive their routine childhood immunizations in time. The Ukrainian immunization schedule is similar to those used in many other European countries (Figure 1). Notable omissions from the routine schedule include vaccinations for pneumococcus, meningococcus, rotavirus, varicella zoster virus, and human papillomavirus. Although immunizations should preferably be offered by public health and primary care organizations, this might not always be possible. When a child presents to the ED, this could be used as an opportunity to offer any catch-up immunizations or signpost to the relevant health organizations. Immunizations should be given according to the schedule of the non-resident country where the child presents, and preferably follow local guidelines. When offering immunizations in the ED, make sure this is accurately documented to prevent duplication or missed vaccinations, and this should be in line with regulations of privacy and data protection. EDs should provide children without an original immunization passport with a replacement immunization record (7). Administered vaccination names might be unfamiliar; translations of common infections in Eastern European languages are available (8).

## COVID-19

There is a considerable risk for an increase in COVID-19 infections amongst the people fleeing Ukraine. Appropriate personal protection equipment mitigating this risk should be worn by health care providers. Overall, an estimated 34% of the Ukrainian population were fully vaccinated against COVID-19 as per February 20th 2022 (9). Vaccinations were recommended for children aged 12 and over in October 2021 (10). Booster vaccines were recommended for anyone aged 18+ in early January 2022, with, as per February 20th 2022, only 2% of the population having received this. Approved vaccines in Ukraine include Moderna, Pfizer/BioNTech, Janssen, Oxford/AstraZeneca, Covishield, Sinovac (11). Refugees and migrants should be included in any regional or national responses to COVID-19, including access to health information diagnostic testing, and medical care (12). Information on COVID-19 testing services, personal protection equipment, quarantine for close contacts and isolation for positive cases (where in force) should be distributed in Cyrillic at EDs and other healthcare facilities in order to contain infection spread.

## Communicable Diseases and Infection Screening

Refugee populations are at higher risk for communicable diseases. Often, these are directly related to exposure during their flight, poor sanitation, and crowded temporary accommodations. In addition, Ukraine has one of the lowest vaccination coverage in the WHO European Region and is below target levels for Diphteria—Tetanus—Pertussis [3rd dose], Polio [3rd dose], measles, and Hepatitis B [3rd dose] (9). Measles immunization levels are the lowest in Europe (13). Outbreaks of cholera, measles, diphtheria, and poliomyelitis are anticipated. The incidence of tuberculosis (TB) is higher in Ukraine than in most other European countries with a significant burden of multidrug resistant TB (27% of new TB cases), and children and young people should actively be screened for TB; similarly, HIV remains a public health issue and a priority communicable disease in Ukraine, and this should be screened for with a low threshold (14). Also, blood borne viruses, such as Hepatitis B and C are seen more frequently in refugee populations (15, 16). In the absence of clinical signs and symptoms that would classify them as infectious according to existing local or public health guidelines, these refugee patients do not need isolation upon arrival in the emergency department. However, it might not be needed to screen for helminths and malaria routinely in this population, unless known high risk. In sexually active or abused young people, consider screening for syphilis and other sexually transmitted infections or referring them to appropriate sexual health services. Consider empiric treatment for intestinal parasites with albendazole in children >2 years and >10 kg. Similarly, check for signs of scabies, headlice, and other soft tissue infections and have a low threshold for treating this. In line with international recommendations, we propose the following infectious diseases screening at first opportunity:

- Perform a tuberculosis screening for latent infection (tuberculin skin test/ interferon-gamma release assays) followed by chest x-ray if either test is positive
- HBV-antibodies (Hbs-Ag, anti-Hbs and anti-HBc) and HCV antibodies
- If immune suppressed: Strongyloides serology
- Low threshold for investigating HIV status

## Nutrition, Dental Health, Vision, and Hearing

Typically, dehydration and malnutrition are frequently encountered medical problems in refugee populations. As the war continues these might become more relevant for children and young people from Ukraine when they visit urgent and emergency care facilities. Consideration should be given to testing for (1) Hemoglobin level to check for anemia and treat iron deficiency; (2) Vitamin D level if at risk for malnutrition and rickets. Mothers with young infants will benefit from easy access to (breast) feeding support teams. All children and young people, and in particular those with dental issues, should be seen by a dentist following their arrival in another European country. Similarly, vision and hearing should be tested in all refugee children and young people, as impairments can heavily impact on quality of life and social and educational integration.

## Trauma

Although European EDs might have some experience in mass casualties following isolated major incidents (17), few, if any, will be familiar with looking after an ongoing stream of acute or delayed presentations of children with war-related injuries. Some of these children will have received rudimentary initial medical or surgical management. It is important to take note of any prior blood transfusions, tetanus vaccination status, and any antibiotics prophylaxis given. Useful resources covering both mass casualty management in the pre-hospital and hospital setting are available via the WHO Academy (18). This also includes information on management of chemical, biological, and nuclear agents. Recent experiences from Israel looking after children with war-related injuries from Syria have shown us that injuries are often complex, needing collaboration of multiple hospital departments. One study described that injury mechanism were most often penetrating injuries, followed by blunt trauma and blast injuries, caused by fragments, blasts, and gunshot wounds. Most common injuries were head trauma and lower extremities injuries (19). Setting up coordinated military-civilian retrieval of severe pediatric warzone trauma in the neighboring countries might improve outcomes (20).

## Mental Health

The war and the resulting displacement and separation from friends and family will lead to a surge of mental health issues in young people who fled from Ukraine. Some of these young people will present to EDs with acute mental health issues, such as intoxications, intentional ingestions, self-harm, suicidal ideation, anxiety, sleeping disorders, and post-traumatic stress disorder (PTSD). For those who pose an acute risk to themselves or to others, usual pathways for mental health conditions should be followed. However, active screening for broader mental health issues will be important, for example by using the internationally recognized HEADSSS (Home, Education/Employment, Activities, Drugs, Sex and relationship, Self harm and depression, Safety and abuse) tool for the assessment of adolescent patients (21) and screening for symptoms of PTSD (Appendix 2). Identifying a local pathway for providing formal mental health assessments away from the ED, and signposting available community services to families are important preparatory steps, recognizing that language barriers will be a major challenge. Of note, screening for symptoms of PTSD in this vulnerable and at risk population is important to decide on the choice of sedative agents when performing procedural sedation in the ED (e.g., avoid ketamine as single sedative agent, and consider additional use of midazolam).

## Social Determinants of Health

We stress the importance of integrating displaced families in local communities urgently, minimizing time away from education, peer support, and social activities. Clinicians working with children and young people in urgent and emergency care will need to be prepared to liaise with primary care and community pediatricians to arrange ongoing care for displaced families. All these children and young people should undergo appropriate and expedited health checks, and appropriate resources need to be allocated for this. Strategies for continuing care after ED visit, and for performing investigations, such as infection screening, and reporting results back to the family and public health organizations should be tailored to regional health systems. Similarly, clear lines of communication with social services will need to be in place, and caregivers should be given information about the role of social services at a national and regional level and about available support in the local community. We foresee that considerable numbers of young people seeking our help will be unaccompanied. When children and young people are accompanied by adults, efforts should be made to establish if these are the legal guardians of the child or young person. Details of the original family composition and current caregivers should be shared with social services. Accompanying adults should be signposted to appropriate health care facilities for their medical problems or routine health checks. There is clear guidance on appropriateness of diagnostic tests for young people with disputed age and the role of pediatricians for age assessments, and there is no role for bone radiographs or dental X rays for age determination in EDs (22).

Finally, we acknowledge the extremely difficult times our colleague pediatricians from Ukraine are experiencing, whether they are still working in their home country, or have made the difficult decision to leave Ukraine. As international societies, we will need to think about pathways for and empower our Ukrainian colleagues who wish to continue their profession outside their home country.

## Data Availability Statement

The original contributions presented in the study are included in the article/Supplementary Material, further inquiries can be directed to the corresponding author/s.

## Author Contributions

All authors are members of the executive committees of the European Society of Emergency Pediatricians and European Academy of Pediatrics, and the statement was written as part of a coordinated response by both international societies. All authors had original input into writing the manuscript and reviewed and approved of the final version as submitted.

## Funding

RN was funded by NIHR ACL award (2018-021-007).

## Conflict of Interest

The authors declare that the research was conducted in the absence of any commercial or financial relationships that could be construed as a potential conflict of interest.

## Publisher's Note

All claims expressed in this article are solely those of the authors and do not necessarily represent those of their affiliated organizations, or those of the publisher, the editors and the reviewers. Any product that may be evaluated in this article, or claim that may be made by its manufacturer, is not guaranteed or endorsed by the publisher.

This article was submitted to General Pediatrics and Pediatric Emergency Care, a section of the journal Frontiers in Pediatrics.
