# Supplementary material for: Update on the Coordinated Efforts of Looking After the Health Care Needs of Children and Young People Fleeing the Conflict Zone of Ukraine Presenting to European Emergency Departments—A Joint Statement of the European Society for Emergency Paediatrics and the European Academy of Paediatrics
Source: Front Pediatr. 2022 Apr 26;10:897803. doi: 10.3389/fped.2022.897803 (PMC9090499; doi:10.3389/fped.2022.897803)
Supplement: Supplementary file 1 [file Data_Sheet_1.pdf]

Appendix 1. Example template for use in emergency departments

REFUGEE 0-18 DATASHEET

МЕДИЧНА КАРТКА ДОГЛЯДУ ЗА ДИТИНОЮ-БІЖЕНЦЕМ У ВІКУ 0-18 РОКІВ МЕДИЧНОЇ СЕСТРИ

(Fill with capital letters /заповніть форму друкованими літерами)

CHILD'S NAME /ИМЯ РЕБЕНКА/ Ім'я дитини	
Child's birthdate<br>*.... day.... month..........year*<br>*Birthplace:*<br>*..............country.......town*	Дата народження дитини?Дата Рождения ребёнка?а<br><br>Дата народження: .... рік .... місяць .... день<br><br>Місце народження/место рождения : .... країна/страна .... населений пункт
CHILD'S PASSPORT (ID card) NUMBER/ НОМЕР ПАСПОРТА(удостоверение личности)РЕБЕНКА/ Номер паспорта(посвідчення особи) дитини	
NEXT OF KIN'S NAME / Имя родственника/ Ім'я родича	
NEXT OF KIN'S PASSORT (ID card) NUMBERHOMEP ПАСПОРТА (удостоверение личности) РОДСТВЕННИКА/ Номер паспорта (посвідчення особи) родича	

**English/ Ukrainian**	**Yes**	**No**
WOULD YOU LIKE HELP WITH ACCOMODATION? /Чик потрібна вам допомога в цьому? Нужна ли вам помощь в этом?	так /да	Ні/нет

Name of legal guardian, (telephone) contact details:<br>Ім'я законного представника, (телефон) контактні дані:<br>Имя законного представителя	
Without legal guardian, accompanying person's name:<br>з незаконним представником, ім'я представника/опікуна:<br>с незаконным представителем, его имя :	
Accompanying person's (phone) contact details<br>Контакти представника/опікуна (телефон):<br>Контакты опекуна/ представителя:	

Child’s symptoms in past 10 days/симптомы за последние 10 дней у ребёнка:

Symptoms/симптомы	Yes	No
fever/температура (>=38°C):	Так/ да	Ні/ нет
lethargy/слабость	Так/ да	Ні/ нет
runny nose/насморк	Так/ да	Ні/ нет
chough/кашель?	Так/ да	Ні/ нет
throat pain / Болить горло	Так/ да	Ні/ нет
dry chough /Сухий кашель	Так/ да	Ні/ нет
wet chough / Катаральний кашель	Так/ да	Ні/ нет
short of breath / трудное дыхание	Так/ да	Ні/ нет
chest pain / грудная боль	Так/ да	Ні/ нет
loss of smell or taste / не чуствовает запах/вкус	Так/ да	Ні/ нет
headache/головная боль	Так/ да	Ні/ нет
muscle ache/мышца боль	Так/ да	Ні/ нет
abdominal pain/ Болить живіт?	Так/ да	Ні/ нет
vomiting/рвота	Так/ да	Ні/ нет
diarrhoea/понос	Так/ да	Ні/ нет

CHILD’S MEDICAL HISTORY/СТАН ЗДОРОВ'Я ДИТИНИ

Does the child have any chronic disease requiring treatment?<br>Чи має дитина хронічне захворювання, яке потребує лікування?<br>Имеет ли ребенок хроническое заболевание, требующее лечения?	Так/ да	Ні/ нет
If yes, please provide details. Якщо так, тоді яке захворювання? Если да , какое?		
Does the child have heart condition? Чи має якісь хвороби серця? Есть ли какие-то болезни сердца?	Так/ да	Ні/ нет
Does the child have diabetes? Чи хворіє дитина на цукровий діабет?Болеет ли ребенок на сахарный диабет?	Так/ да	Ні/ нет
Does the child have asthma? Чи є в дитини Астма?Есть ли у ребёнка Астма?	Так/ да	Ні/ нет
Does the child have epilepsy? Чи є в дитини епілепсія? Есть ли у ребёнка епилепсия?	Так/ да	Ні/ нет
Does the child take any regular medication? Please list them. Чи є ліки, які регулярно приймає , якщо так , то які ?Есть ли лекарства , которые регулярно принимает, если да , какие?	Так/ да	Ні/ нет
Does the child have enough medication supply? Чи маєте достатню кількість ліків? Есть ли у вас сейчас достаточно лекарства?	Так/ да	Ні/ нет
Does the child have any allergy for any medication? Чи є алергія на ліки? Есть ли аллергия на некоторые лекарства?	Так/ да	Ні/ нет
If yes, please list he medication and the reaction. Якщо так, на які саме? Если да , на какие именно?		
Does you have any food allergy? Чи є в дитини алергія на будь які харчові продукти ? Есть ли у ребенка аллергия на какие-то продукты питания?	Так/ да	Ні/ нет

If yes please list the allergens. Якщо так, на які саме?Если да , на какие именно?		
Does the child have lactose intolerance? Чи є в дитини непереносимість лактози? Есть ли у ребёнка непереносимость лактозы?	Так/ да	Ні/ нет
Does the child have gluten allergy? Чи є в до глютену?Или глютену?	Так/ да	Ні/ нет

RECEIVED IMMUNISATION /ОТРИМАНІ ЩЕПЛЕННЯ/ПОЛУЧЕННЫЕ ПРИВИВКИ

Did the child receive measles vaccine?	Чи має дитина щеплення від корі?Есть ли у ребенка прививка от кори?	Так/Да	Ні/Нет
Did the child receive CoVid-19 vaccine?	Чи має дитина щеплення від Ковід?	Так/Да	Ні/Нет
Did the child have CoVid-19?	Чи хворіла дитина на Ковід?Болел ли ребенок Ковидом?	Так/Да	Ні/Нет
If yes, when?	Якщо так, коли? Если да , когда?		

When and which vaccine did the child receive?	Коли та які прививки отримували?	**Yes**	**No**
BCG:	BCG:	Так/Да	Ні/Нет
Di-Per-Te:	Di-Per-Te:	Так/Да	Ні/Нет
Hib:	Hib:	Так/Да	Ні/Нет
IPV or OPV (polio):	IPV чи OPV:	Так/Да	Ні/Нет
Pneumococcus:	Pneumococcus:	Так/Да	Ні/Нет
Morbilli:	Morbilli:	Так/Да	Ні/Нет
Rubeola	Rubeola	Так/Да	Ні/Нет
Mumps:	Mumps:	Так/Да	Ні/Нет
Meningococcus B	Meningococcus B	Так/Да	Ні/Нет
Meningococcus C.	Meningococcus C.	Так/Да	Ні/Нет
Rotavirus enteritis	Rotavirus enteritis	Так/Да	Ні/Нет
Varicella:	Varicella:	Так/Да	Ні/Нет
Hepatitis A:	Hepatitis A:	Так/Да	Ні/Нет
Hepatitis B:	Hepatitis B:	Так/Да	Ні/Нет

The child needs to be admitted to the hospital	Необхідно залишитись в лікарні на обстеження. Нужно будет остатся в больнице на обследование		
Mother allowed to stay with the child in the hospital.	Мамочка може залишитись з дитинкою. Мамочка может остатся с ребёнком		
Child will receive an intravenous drip.	Отримаєте крапельницю. Получите капельницу		
Laboratory test are necessary (blood, urine)	Необхідно здати аналізи (кров, сеча) Нужно будет здать анализы (крови, сечь)		
Blood sugar test is necessary	Необхідно здати аналізи на цукровий діабет. Нужно здать анализы на сахарный диабет		
X-ray examination is required	Необхідно зробити рентген знімок. Нужно сделать рентген снимок		

Ultrasound examination is required	Необхідно УЗД. Нужен УЗД		
Child could be discharged and need to take medication.	Не потрібно залишитись в лікарні, але повинні приймати ліки.Не нужно оставаться в больнице, ну вы должны начать принимать лекарства		
Will need to take …tablets/mls …. Times per day.	Приймання ліків/ Прием лекарств ……...в день ....штук		
You need to bring the child back for review in ….. days to….. ward	До лікаря повернутись через/ К врачу вернутся через ………. днів/дней,<br><br>на ..... ………………………........ У відділення/ в отделение		

Appendix 2. HEADSSS (acronym) assessment in young people.

Legend: resources: https://dontforgetthebubbles.com/mental-health-screening/ and https://teachmepaediatrics.com/community/holistic-care/headsss-assessment/

When performing a HEADSSS assessment with young people ask about:	Example questions:
Home	Where do you live?<br>Who lives at home?<br>Do you have your own room?<br>Do you fight with anyone at home?<br>Is there anyone you particularly get on with?<br>Who do they turn to when upset?
Education & Employment	Do you go to school/college?<br>What subjects do you enjoy? What subjects don't you like?<br>Do you have a job? What sorts of hours do you work?<br>What's the best thing about working? What don't you like so much?<br>What would you like to do in the future?<br>Who are your friends at school/work?<br>Does anyone bully you?
Eating and Exercise	Do you worry about the shape of your body or your weight?<br>What do you like or not like about your body?<br>Do you try things to manage your weight?<br>Have you ever made yourself throw up to lose weight?

	Are any of your family or friends worried about your weight or your attitude towards your body?
Activities	What do you do in your spare time?<br>How do you relax? What do you do for fun?<br>What do you like doing with your friends?<br>Do you participate in any sports/physical activity/exercise?<br>How much TV do you watch? What are your favourite shows?<br>Some teenagers tell me they spend a lot of their free time online – what do you use the internet for?
Drugs/Drinking	Some people your age try smoking, alcohol and drugs, is that something you've experienced?<br>How much? How often?<br>What does taking drugs/drinking alcohol do for you?<br>Where do you get the money?<br>Where/who do you get your drugs from?<br>Does your alcohol use cause you any problems?<br>Are you interested in cutting down or stopping?<br>Is there anything I could do to help you with that? Would you like to see someone about that specifically?
Sex	Have you ever been involved in a romantic relationship?<br>Have any of your relationships been sexual ones?<br>Are you seeing anyone at the moment?<br>How do you see yourself in terms of sexual preference? Are you interested in boys, girls, both, or not sure?<br>Have you ever had an experience in the past that you did not feel comfortable with?<br>What contraception are you using?

	How do you handle intimate relationships? Do you feel pressure to go along with things that you'd rather not do?<br>Does your partner give you things in exchange for sex or other physical acts?
Self-harm, depression & suicide	How is your mood at the moment?<br>Do you ever feel sad or stressed? What do you do about that?<br>What sorts of things make you feel low/sad/stressed?<br>Have you ever thought about hurting yourself?<br>Have you acted on those thoughts?<br>Have you thought about ending your life?
Safety (including social media/online)	Do you ever feel unsafe?<br>Is there anyone in your life that you don't feel safe around?<br>Is anyone doing things to you that you don't want them to? What sort of things?<br>Does anyone put pressure on you to do things you don't want to do?<br>Have you ever deliberately harmed or injured yourself?<br>Have you ever put yourself in unsafe situations – risky driving, unsafe sex, that kind of thing?<br>Do you belong to a group or gang?<br>Have you ever been in trouble with the police?<br>Is there anyone you can talk to about these things?
